# Chicago Medical Society

**Published:** 1869-05

**Authors:** 


### CHICAGO MEDICAL SOCIETY.

Friday Evening, March 5, 1869.

The Society was called to order, and in the absence of President Marguerat, Dr. W. Kersham was appointed to fill the chair *pro tem.*

The Secretary read the minutes of last meeting, which were duly approved.

Drs. Groesbeck and Wickersham recommended Dr. J. R. Groesbeck for membership. Referred to Board of Censors.

The Board of Censors reported favorably in the case of Dr. Lyman Ware. Society proceeded to ballot, Dr. Ware being unanimously elected a member of the Society.

Dr. Paoli asked permission to read a letter from a member of the Legislature in relation to the Medical Bill, to the effect that the bill would not pass this session for want of time, as it had at that time not been reported on.

Dr. Groesbeck remarked that he was sorry the Society had taken any action to promote the passage of the bill, and hoped they would cease all further action in the matter.

Under call for report of cases, Dr. Paoli reported case of a lady on Erie St., who supposed herself suffering from inflammation of the bowels. Dr. found no inflammation, but retroversion of the uterus. The woman had some accident five years ago. Lately she has suffered a great deal from leucorrhœa, which the Doctor thinks is the great cause of the retroversion. Under the use of tonics, iron, etc., the patient is doing well.

Dr. Paoli asked of the Society what diseases were most prevalent in the city.

Dr. Davis reported the cases of two girls, aged respectively 10 and 15 years, who had suffered from scarlatina in a mild form. In one week, the youngest complained of pain in the back. There was general anasarca, continuing four days. Urine scanty and high colored. She took bitartrate, nitrate, and acetate of potassa, and digatalis. Notwithstanding only one passage of urine was effected for 24 hours, which was half blood. This was soon followed by symptoms of giddiness, dilatation of the pupils, and quick, successive convulsions, continuing for 18 hours.

Everything taken into the stomach was rejected. Bromide and iodide of potassium was given without effect, together with vapor baths aud fomentations. Still convulsions continued. I ordered nitrate of potassa and calomel, each 5 grs., to be given every hour until two doses were taken, then the interval lengthened to every two hours, alternated with a mixture of chloroform and tinct. cannabis Indica. Four powders were given before the bowels were moved, evacuation being copious. Then withdrew the calomel and gave nitrate potassa and pulv. Doveri āā gr. ij., every four hours, continuing the chloroform and cannabis Indica. Child is now well.

In the other case, the girl of 15 years, I could get nothing to act on the bowels until I finally prescribed three gtts. oleum tiglii, part of which was rejected. It, however, produced a copious evacuation, which relieved her. For one week she seemed quite well, when she was again seized with violent convulsions, which continued for 24 hours. Dr. Byford saw her and ordered a vapor bath and some other remedies. It is now one week since she has had a convulsion. These are the only cases of scarlet fever that have been followed by anasarca and convulsions that I have met with for a considerable period of time. As regards the prevalence of disease, the Doctor stated that there is a great deal of pertussis, but not sufficient scarlet fever to be called an epidemic. Has noticed some cases, however, which assumed a diphtheritic form.

Dr. Groesbeck reported fatal cases of scarlet fever, the first a little boy of 3½ years. At an early stage of the disease there appeared on both tonsils white patches which extended to the mouth. The patches, however, soon cleared off, and I looked for convalescence. After one week had elapsed, the tonsils began to swell, and in due time I opened one tonsil, considerable pus escaping. In a day or two later there was severe hemorrhage from the sloughing surface of the tonsil, and the child died.

The other case, a boy of 4½ years, was taken with the same symptoms, the same exudation appearing on the tonsils and in the mouth. When convalescence was expected in this case they became swollen and edema of the lungs supervened.

Dr. Paoli thinks that when you find blood present in the urine in acute Bright's disease following scarlatina, that it is a favorable symptom. The Doctor wished to know if any member had used cannibis Indica alone as an antispasmodic.

Dr. Davis says there was no effects of antispasmodics in the cases referred to until after free elimination had been produced, but thinks the cannibis Indica would have acted favorably alone.

Dr. Davis says he saw Dr. A. A. Dunn four days before his death, and that Dr. Andrews had seen him several days previously, as he was attending him. Says that Dr. Dunn seemed to think he was suffering from neuralgia in the cicatris, on his forehead, although the pain was somewhat diffused. The Doctor seemed wearied and tired, and had been taking morphine and quinine while going about. Nearly a week before his death, he said he felt as though his end was coming. I visited patient that day. He grew rapidly worse. Pulse not accelerated. Very moderate increased heat of head. Went steadily, although quite rapidly, into a dull, drowsy condition, but if roused would talk. Pupils a little dilated. When I was again called I could not rouse him sufficient to converse, although he seemed to recognize me. Pulse soft and compressible. Pupils contracted and but little affected by light. Bowels inactive. Gave an enema. Red and congested condition of the nases and fauces, but throat was not swollen. He continued to sink into a stupor, and the third day after I saw him he died. There is no doubt in my mind but what there was disease of the anterior lobes of the brain, as we could not get him to swallow, the second day after I saw him. At the time of death there seemed to be edematous infiltration about the glottis. Dr. Davis also spoke highly of the deceased, and recommended that a committee of three be appointed to adopt resolutions and send a copy to the family of the deceased and copies to the city papers.

Dr. Fitch said that he has been acquainted with Dr. Dunn for twenty years, and all that Dr. Davis has said is true. Remarked that the Doctor did not serve in the army as a surgeon but as a Captain in the line. During the service he received a shell wound of the forehead, which continued to discharge for two years and only healed about one year ago. Dr. F. was also inclined to think the wound was the predisposing cause of his sickness, as there was a fracture of the cranium.

Dr. Holmes said Dr. Dunn gave a long report of his own case to the Society some time ago, and stated that spicula of bone had been discharged from the nostril.

Dr. Fitch said that he thought Dr. Dunn had seemed depressed and dull ever since he received the wound, not appearing like the same man to him.

On motion, a committee was appointed, consisting of Drs. Davis, Fitch and Groesbeck, to report resolutions in the case of Dr. Dunn at the next meeting of the Society.

After transaction of some miscellaneous business the Society adjourned.

---

CHICAGO, FRIDAY EVENING, March 12, 1869.

Society was called to order, President Marguerat in the chair.

Secretary Macdonald read the minutes of last meeting, which were duly approved.

Board of Censors reported favorably in the case of Dr. J. R. Groesbeck. Society proceeded to ballot, Dr Groesbeck being unanimously elected a member of the Society.

The Secretary read the resignation of Dr. J. E. Ray, which after some discussion was duly accepted by the Society.

Then proceeded to the discussion of the "Hypodermic Application of Medicinal Substances."

The discussion was opened by Dr. Ingalls, who gave an interesting history concerning its discovery. Dr. Wood, of Scotland, in 1850, introduced this method of applying medicines, but it has been proven that Dr. Brainard, of Chicago, used a syringe constructed on the same principle, and manufactured for him by Tieman, as early as 1845, and used it in the treatment of spina bifida, hydrocephalus, ascites, etc., and that he wrote several articles on the subject about that time. Hence we can justly say that the system of the hypodermic application of medicines originated in Chicago. The Doctor also considered the different complaints in which it was most applicable.

Dr. Marguerat said that he was glad to hear Chicago had a claim to such a valuable discovery, but did not think it would supercede the administration of drugs by the old way. Says he noticed a case of death from erysipelas, following a large injection of quinine, but thinks there is no doubt but what it is of great service in the treatment of neuralgia, dysmenorrhœa, etc., and believes its effects more permanent than when otherwise administered. Said he was called in the case of a woman who was suffering from hysterical insanity, the result of ill treatment by her husband. She had been a raving maniac for three weeks; could not give her medicine, as it took three or four to hold her in bed. He injected morphine, and in five minutes the patient passed into a quiet sleep.

Dr. Paoli remarked that he had no doubt but what the time will come when the hypodermic application of medicine will be approved by the profession generally. In cholera time, said he derived more benefit from the administration of morphine in this way than by any other. Has had no experience with other alkaloids.

Dr. Foster asked what proportion of morphine was most applicable for hypodermic action.

Dr. Fitch said he had but little experience in this mode of treatment, except as recommended by Dr. Brainard in the treatment of varicose veins and hydrocele. Generally introduces 15 or 20 drops of the liquid, first pinching up a fold of the skin before inserting the point of the instrument. Says Dr. Anstie, in *Braithwaite's Retrospect*, considers atropine of great value when the disease is located in the pelvis or abdomen, and strychnia in gastralgia. Dr. F. reported a case of abortion which occurred two months ago, when the patient was in great pain, restless and uneasy, and could take no food. Gave $\frac{1}{75}$ gr. atropine with great success; after second dose was introduced there was dilatation of the pupil.

Dr. Davis remarked that he had not much experience in the use of the hypodermic injection. Used it in one case of facial neuralgia with permanent benefit. Patient has had no attack for a year. Used it in several other cases with temporary benefit. Thinks when the solution contains too much acid then it is apt to be followed by ulceration or abcess.

Dr. Ingalls thinks that what Dr. Davis has said is true, and is of the opinion that all acids should be discarded from injections, always using distilled water. (Magendie's solution morph. gr. xvj.; aqua ℥j.)

Dr. Bogue thinks there is great benefit from the addition of from 1-40 to 1-80 of a gr. of atropine to about a-half of a dose of morphine, as it extends the effects of the anodyne from 12

to 16 hours. Thinks this method very useful given in the cramps of cholera.

Society passed to miscellaneous business.

The committee appointed to report resolutions in the case of Dr. Dunn submitted the following, which was duly accepted by the Society:—(See Resolutions in April number.)

Society adjourned.

---

FRIDAY EVENING, April 16th, 1869.

Society called to order by the President, Dr. R. G. Bogue.

Reports of cases being the order of the evening, Dr. N. T. Quales reported the following interesting case of rupture of uterus:—

March 9th, 1869, at two o'clock P.M., I was called to tend Mrs. L., a strong, healthy Irishwoman, aged 28, in her third confinement—two previous having been instrumental deliveries—told me she had been sick since five o'clock in the morning; pains having been strong and regular; membranes ruptured half an hour before my arrival, and about 15 or 20 minutes later (the pains having continued with increased severity) she felt something "give way," and the pains almost instantly ceased.

On examination, I found the os uteri fully dilated, the cord down, but no parts presenting. By introducing the hand, I found the promantory of the sacrum unusually prominent, and by carrying the hand farther, it came in contact with the umbilicus, and I made out the position as transverse, the abdomen presenting—the head to the right, and the feet to the left, side of the mother. In passing my hand (right) round in order to get hold of the feet, I found a longitudinal rupture of the posterior wall of the uterus, above the promontory of the sacrum, about 2½—3 inches in length, with intestines protruding. My feelings at this discovery can better be imagined than described. I despatched a messenger for my friend Dr. Paoli.

With the conviction that immediate action offered her the best chance, I decided to turn and deliver at once. I brought down the left foot, and, by gentle traction, succeeded in delivering her, in course of 15 or 20 minutes, of a fullborn, healthy male child—apparently stillborn, yet, after some patient effort, I had the satisfaction of seeing vitality restored.

By gentle traction on the cord, the placenta was expelled in about three inches. There was now some considerable flooding. I at once gave ʒij. of the fl. extract of ergot (Duffield's), introduced my hand and replaced the protruding intestines, and, by friction and pressure over the abdomen, caused firm contraction of the uterus before I withdrew my hand. In course of 15 minutes, I repeated the ergot, in order to obtain continued contraction; and having succeeded in this, I applied a moderately tight bind and napkin to the ulva—waited another half hour—the contraction of the uterus continued. I gave gr. ij. of opium, and left orders to call me if anything unusual should occur. At eight o'clock in the evening, I called and found the uterus somewhat dilated, the patient otherwise comfortable. Ordered gr. ij. of opium at once, and to be followed with gr. j. doses of opium every two or three hours, if she was awake.

March 10th, at eight o'clock A.M., I found her feverish and uneasy. She had slept about three hours during the night, and passed urine twice. Pulse 112 per minute; respiration somewhat labored; tongue dry; considerable tympanites and tenderness about the uterine region; lochial discharges suppressed. Ordered tinct. verat. vird., gtt. 4, every three hours, and pulvis opium and dydrorg. submureas, of each, gr. j., every two hours, with turpentine stupes over the abdomen: saw her at noon, when she was more comfortable. At eight o'clock in the evening, the pulse was 108 per minute; the tenderness about the abdomen subsided. Ordered gr. ij. of opium, at bedtime.

March 11th, at eight o'clock A.M., pulse 106 per minute; no great pain; had slept several hours during the night, and taken some nourishmeut. Treatment continued, with longer intervals between the doses. Also, injection into the uterus of solution of acid carbol., gtt. vj. to the ℥j. of warm water, three times a day.

March 12th. Symptoms much aggravated; pulse 120 per minute; tongue dry; lympanites and tenderness increased; had passed a restless night. Ordered blister, 12×12, over the abdomen, to be left on for six hours. Internally, I ordered quinia sulph., gr. j.; pulvis opium, gr. ss., every four hours, to alternate with tinct ferrsh, gtt. xx. On removing the blister, a large, warm flax-seed poultice was applied to the abdomen, and a full anodyne at night.

March 13th. Much improved; little pain besides the soreness from the blister; lympanites greatly subsided; pulse 112 per minute; tongue moist; bowels moved for the first time since confinement; locheal discharges reestablished; took considerable nourishment during the day.

March 14th. Improving; pulse 90 per minute; tongue moist; no pains, and but little lympanites: treatment continued.

March 27th. Sits up, and can walk across the floor. Secretion of milk liberal.

At the present writing, April 14th, 1869, both mother and child are doing well; the mother performs her ordinary household duties, yet complains of occasional soreness over the abdomen.

Dr. G. C. Paoli, in remarking on the foregoing case, gave the following statistics of ruptures of the uterus:—

In the Kingdom of Wurtenburg, in 219,535 births was observed six ruptures of the uterus, being only one in 36,539. Madam La Chapel observed in Paris Hospital only one in 20,000 births.

Professor Jocery Elipse observed two ruptures in 20,056.

Dr. Erringman, of Prague, from 1827 to 1833, observed seven ruptures in 18,085 cases.

Dr. Cedershold, of Sweden, from 1830 to 1831, observed two ruptures in 2334. Churchill, of England, in 42,768 there was 75 cases, making 1 in every 657 which occurred in Dublin.

Verbal reports of cases were made by Drs. Groesbeck, Paoli, Mitchell, and others.

Dr. T. D. Fitch, one of the surgeons to the Cook County Hospital, reported a case of death from the inhalation of chloroform, which occurred that day at the Hospital.

The patient was an adult, native of Sweden, and a laborer. Several months since he suffered a severe injury of his foot and ankle, by a waggon-wheel passing over it. The injury had resulted in extensive destruction of soft parts by suppuration, and caries of the bones of the ankle.

He was admitted to the Hospital only a few days since; and a consultation of the surgeons of the institution resulted in the decision that amputation was necessary. The patient had been kept on good diet and tonics during the short time he had been in the Hospital, and had taken a glass of wine immediately before entering the operating room. No disease had been detected in the organs of respiration or circulation; and the patient was himself anxious to have the operation performed. The chloroform was administered on a napkin, held over the nose and mouth, not so close as to prevent the free access of atmospheric air.

When the inhalation had progressed from one to two minutes, and ten or twelve inspirations had been taken, an unusual sound was noticed, and the napkin immediately removed. A slight tremor of rigidity or spasm passed over the muscular system; three or four slight efforts at inspiration took place at long intervals, and then ceased entirely with complete muscular relaxation. The heart, however, continued to beat feebly for more than half an hour after the respiration ceased. The most strenuous efforts were made to revive the patient by artificial respiration, and otherwise, for more than one hour. The account of Dr. Fitch was corroborated by Drs. Bevan and Bogue, who were present and assisted in the efforts to restore the patient.

A minute and careful *post mortem* was made the following day, but no disease of the organs of circulation or respiration were found, and no congestion or even fulness of the vessels of the brain.

After the transaction of some miscellaneous business the Society adjourned.

